# Supplementary material for: PrEP in the key population community: A qualitative study of perspectives on pre-exposure prophylaxis by gay, bisexual and other men who have sex with men and female sex workers in Kigali, Rwanda
Source: PLOS Glob Public Health. 2025 Apr 24;5(4):e0004538. doi: 10.1371/journal.pgph.0004538 (PMC12021139; doi:10.1371/journal.pgph.0004538)
Supplement: S1 Text — (PDF) [file pgph.0004538.s001.pdf]

PrEP Uptake and Retention among Key Populations in Rwanda and DRC

In-Depth Interview Guide for Study Participants

Introduction

Welcome and thank you for joining us! My name is (**introduce facilitator**). I work with researchers from the Albert Einstein College of Medicine in New York, RD Rwanda in Kigali and the School of Public Health at the University of Kinshasa. We are conducting a study on Pre-Exposure Prophylaxis (PreEP) among members of such communities as men who have sex with men, sex workers, transgender people, and others in the DRC and Rwanda. With me today is (**introduce note taker**) who will be helping me to take notes of important issues during our conversation. We have requested you to join us today so we can learn fromyour experiences and hear your perspectives on a range of issues about Pre-Exposure Prophylaxis (PreEP), which is a medication that you can take to prevent getting HIV from sex or injection drug use. All the data you provide will remain completely confidential.

Today, the focus of our conversation will be on three key areas, which include the following:

1. We will talk about your awareness and knowledge about PrEP as well as your personal history with PrEP;
2. We will discuss the structural and logistical barriers to accessing PrEP;
3. We will talk about knowledge, attitudes and behaviors of people in this community to using PrEP.

We expect to be together for a total of 60-90 minutes. I would also like to inform you that we will be recording our conversation on a voice recorder so that we do not forget all the important information that you will share with us today. I hope you don't mind having our conversation recorded, but if you have any objections to being recorded, please let me know and we'll turn off the recorder.

Please note that participating in this interview is entirely voluntary. You may choose to leave now or any time during the interview. You should be aware that you do not have to answer any questions we ask you if you are not comfortable.Your decision will not affect your rights now or in the future in anyway. We have made arrangements to reimburse you for the cost of your travels to participate in this meeting. Please feel free to ask any question now or after the discussion. **Before we proceed, we willneed you to complete the informed consent sheet, which will indicate that you are here of your own free will and voluntarily consent to share your opinions, experiences, andperceptions with us.**

Complete the informed consent form

We will now begin the discussion.

I. Descriptive Data on Clinical Health Behavior:

1. What is your age?

2. What is your education? (Elementary school/no school ; secondary school ; college/university)

3. What is your marital status ? (Single ; cohabiting ; married ; divorced ; separated ; widowed)

4. Do you consider yourself a man, woman, gransgender, or other? (man ; woman ;

trangender man (MTF) ; transgender woman (FTM) ; other
5. What was your sex at birth? (male ; female ; other, refused to answer)
6. What is your sexual orientation? Are you attracted to and have sex with Men only ; women only ; men and women ?
7. How many different sexual partners have you had in the past 6 months?
8. Have you injected any drug other than those prescribed for you in the past 6 months? [Probe: can you share which drug(s) you have injected in the past 6 months? About how many times monthly would you say that you've injected this/these drug(s)]
9. Have you been diagnosed or treated for a sexually transmitted infection in the past 6 months? [Probe: which STIs have you been diagnosed with?]
10. Have you been given or received money, drugs, or anything that you value in exchange for sex in the past 6 months? [Probe: About how many times monthly, would you say this exchange occurred?]
11. Did most part of your income come from sex work in the past 6 months?
12. Many people have challenges using condoms during sex. Can you tell me about challenges that you have using condoms?
13. When did you last have an HIV test (approximately) ?

We are now going to start our interview that will be recorded which is why I am going to start recording.

II. PrEP Awareness, Use and Adherence

1. Have you ever heard of PrEP?

If the answer is **YES**	If the answer is **NO**
a. What do you know about PrEP? [Probe(s): name, purpose, use]	**Let me briefly explain what PrEP (pre-exposure prophylaxis) is. This is a medicine people can take to prevent getting HIV from sex or injection drug use. When taken as prescribed (one pill every day), PrEP is highly effective for preventing HIV. PrEP is currently becoming more available in DRC and Rwanda; this is the reason we are asking you about it.**
**Let me briefly explain what PrEP (pre-exposure prophylaxis) is. This is a medicine people can take to prevent getting HIV from sex or injection drug use. When taken as prescribed (one pill every day), PrEP is highly effective for preventing HIV. PrEP is currently becoming more available in DRC and Rwanda; this is the reason we are asking you about it.**	a. When you hear the description of PrEP, does it sound familiar to you?

b. How did you first hear about PrEP? [Probe(s): when, where, who (from a health care provider, friend, community member, etc.)]	If **yes,**<br>b. What else do you know about PrEP outside of the description shared? [Probe(s): name, purpose, use]

2. Have you ever used PrEP?
<u>If YES, skip to 2b. If NO (have never taken PrEP), ask 2a. and skip to Section III.</u>

2a. Why haven't you used PrEP? What helped you decide not to take PrEP?
2b. If YES, [Probe(s): why? What helped you decide to take PrEP? How long have you been using it?]
2c. Are you still using PrEP? [Probe(s): why or why not?

3. Experiences using PrEP

**<u>For those that still use PrEP:</u>**	**<u>For those that have stopped taking PrEP:</u>**
a. Please tell me about your experience using PrEP [Probe: including perceptions, side effects, efficacy, length of time (month/year), formulation/dosage, frequency of use, oral or injectable, etc.]	a. Please tell me about your experience using PrEP [Probe: including perceptions, side effects, efficacy, length of time (month/year), formulation/dosage, frequency of use, oral or injectable, etc.]
b. What were the most important factors when you decided to use PrEP?	b. Since you are no longer on PrEP, please tell me about your decision to stop using PrEP [Probe: feedback from peers, partners and providers; biggest challenges to stop using PrEP, side effects, cost, no perceived need to keep taking it, stigma, uncomfortable with having a doctor aware of your sex life, etc.]<br>c. If side effects, probe: What side effects did you have?
d. Many people have tried to use PrEP but then stopped using it. What helps you to continue using it? [Probe: peer support and/or dedicated group-based support, not needing to pay, not having to go to their regular doctor for PrEP, using a pill box, taking them at the same time as another activity (e.g.	d. Do you think you'll use PrEP again in the future? [Probe: what resources would you need? more encouragement from health providers, the request of a partner or loved one, new partner, free or reduced cost PrEP, more timely information

while washing face, brushing teeth, etc.), setting alarms, etc.]	about PrEP, access to community health workers to help navigate the health system and/or support their PrEP adherence, etc.]
e. Have you told anyone that you take PrEP? [Probe: What was this experience of sharing this information like?]<br>f. If you have not disclosed: Would you tell anyone? Who would you tell and why?	e. Have you told anyone that you took PrEP? [Probe: What was this experience of sharing this information like?]<br>f. If you have not disclosed: Would you tell anyone? Who would you tell and why?

III. Willingness to use PrEP

For those who have used PrEP: skip to Question 2.

**For those who have never heard about PrEP:**	**For those who have heard about PrEP, but never used it:**
1.<br>a. In other countries where PrEP is available, we find that it helps people take control of their sexual health, eliminates worry and stress related to HIV because it protects people against HIV: Does taking PrEP seem like something you would be interested in? Why or why not?	1.<br>a. Would you be willing to use PrEP if you were offered it by your doctor or other health care providers? Why/why not? Please explain.
b. **If YES:** Do you think it can be useful for you and your health? Why/why not do you think so?<br><br>**If NOT:** What about PrEP does not appeal to you? [Probe: Immediate side effects, such as reduced appetite, nausea, headache and fatigue., long term side effects, discomfort, stigma, others? etc.]	b. **If NOT:** Tell me why you would not want to use PrEP if it is offered to you [Probe: the fear of side effects, frequency of dosage, lack of perceived risk of HIV, stigma, etc.]

2. (Ask everyone) Would you recommend PrEP to anyone? [Probes: Why? Why not? Who would you recommend it to?]

IV. Logistics, Costs and Access to PrEP

1. What are the biggest challenges in accessing PrEP? [Probe: Stigma, lack of trained

personnel, negative attitudes to key populations among health care providers, policy and practical issues around PrEP availability and accessibility, etc.]

2. Where would be the best place to pick up PrEP, in your opinion ? [Probe: Health Facilities, Pharmacies, Community Distribution Centers, Peer Educators, Anonymous sources, etc.]
 a. Probe: why would this location be better than others?

3. One option that we are considering for PrEP and sexual health care would be to have these available in a community-based center that provides services for LGBTQ and sex worker communities, instead of a health center of medical clinic. What are your thoughts about this as an option? What would you like or dislike about it?

4. What other things could be done that would make it easier for you and others to be able to access and use PrEP?

V. Community Knowledge, Attitudes and Motivations to Use PrEP

1. How common is PrEP use among people like you? [Probe(s): Why or Why not? Do people disclose when using PrEP?]
2. How do you think PrEP could be beneficial to members of your community? [Probe: Why or why not? Who do you think would benefit most from using PrEP?]
3. What would be some challenges to PrEP use among people like you (your community – e.g., FSW, MSM, etc.]? [Probe(s): stigma, access, availability, how do you suggest that we address these challenges]

Closing:

1. Is there anything you would like to talk about that we have not covered today?
2. How comfortable were you to talk about all this with me today? What things (if any) were you uncomfortable to talk about?

Thank you so very much for your time as well as the thoughts, experiences, perceptions and opinions you shared with us. We have come to the end and we want to express our gratitude for thetime you have spent with us. We will be available to answer your questions if you have any.
