# Supplementary material for: PrEP in the key population community: A qualitative study of perspectives on pre-exposure prophylaxis by gay, bisexual and other men who have sex with men and female sex workers in Kigali, Rwanda
Source: PLOS Glob Public Health. 2025 Apr 24;5(4):e0004538. doi: 10.1371/journal.pgph.0004538 (PMC12021139; doi:10.1371/journal.pgph.0004538)
Supplement: Attachment — Submitted filename: Review-PrEP in the key population community.pdf [file pgph.0004538.s003.pdf]

Review: Manuscript Number: PGPH-D-24-02716

This well-written manuscript presents a qualitative analysis of cisgender female sex workers and gay, bisexual, and other men who have sex with men in Kigali, Rwanda. Although many of the findings have been previously published, and it would have been preferable to avoid the comingling of qualitative data from two distinct populations since research has identified variability in their health experiences, the paper is noteworthy for exploring collective antiretroviral protection as a sexual health strategy. A strength of the study is that participants were recruited from facility and community settings and included current, former, and never PrEP users. The PrEP perspectives of never-users are well described in the literature; thus, including PrEP re-initiators would have been more interesting, given how users cycle on and off PrEP. Given that intersecting stigmas are recognized as a critical and complex barrier to the uptake of evidence-based HIV prevention interventions, it would have been beneficial to organize the Results (third theme) and Discussion in line with the Socio-Ecological Model, which the authors indicate they referenced in designing the interview guide. I have a few comments which the authors might find helpful.

1) Abstract and Introduction—The authors state that "limited data exist describing experiences and preferences of GBMSM and FSW with respect to accessing and using PrEP" as a rationale for their study. However, a PubMed search (MSM AND PrEP AND Qualitative AND Africa) revealed 23 studies, suggesting their PrEP experiences have been amply described in the literature.

2) Introduction and Methods: Please clarify that this is a study of oral PrEP, given the availability of PrEP rings and injections as alternative formulations.

3) Methods: Participants were recruited from June-July 2022 but interviewed from November-December 2022. What accounts for this five-month gap? Did all who indicated interest in the study eventually participate in interviews, or did some drop off or could not be reached? Were all participants who were approached interviewed, or did some decline?

4) Methods: Line 111 refers to an interview guide. Were separate interview guides used for FSW and MSM? If not, this should be acknowledged as a limitation as these are distinct sub-populations.

5) Results, Table 1. Six MSM had reported sex with both women and men, but marital status is not reported. Were these MSM married to women to fulfill societal obligations to have children and to avoid being outed, as often happens in this setting?

6) Results, lines 163-170: This text is unrelated to the social networks theme and can be moved to after the sentence ending on line 159.

7) Results–Quantification of qualitative research. It is tempting to describe qualitative data using quantitative terms, e.g., "a high level of knowledge about PrEP" (how was this measured?), "a substantial number of participants," or "tremendous amount of anticipated and enacted stigma" (how was this measured?) but this is best avoided.

8) Discussion: The authors state in the Methods that the socioecological model was used to explore individual and structural PrEP barriers and facilitators. However, this is not

evident in the Results (lines 352-367), which do not appear to discuss the findings through the lens of individual, interpersonal, intersectional, or structural stigma.

9) Discussion: Whereas stigma and discrimination in healthcare settings were described in the Results, there is no mention of gender sensitivity training for providers as a possible solution. Additionally, the paper does not mention sex work stigma (sex work is criminalized in Rwanda), particularly for GBMSM who sold sex, as per Table 1.

10) Discussion: Generalizability – avoid the temptation to generalize findings from FSW and MSM in one urban setting by saying your "data suggest that offering options for PrEP care would be well received by key populations in SSA."

11) Language: The NIAID HIV Language Guide recommends not using the term MSM because it is stigmatizing. Gay, bisexual, and other men who have sex with men (GBMSM) is preferred. Moreover, Table 1 indicates that some of these men were bisexual, which speaks to the limitations of describing this population as MSM.

<u>Other comments</u>

12) Line 59: Clarify that the population under study is cisgender FSW and MSM. Currently, the reader only finds this out after seeing sex at birth data in Table 1.

13) Lines 87-88: FSW and MSM were defined in the Introduction and do not need to be written in full in the Methods.

14) Lines 148-149: Please provide a citation for the COREQ guidelines.

15) Line 382: The preferred term is "serodifferent" not serodiscordant.
