# Supplementary material for: PrEP in the key population community: A qualitative study of perspectives on pre-exposure prophylaxis by gay, bisexual and other men who have sex with men and female sex workers in Kigali, Rwanda
Source: PLOS Glob Public Health. 2025 Apr 24;5(4):e0004538. doi: 10.1371/journal.pgph.0004538 (PMC12021139; doi:10.1371/journal.pgph.0004538)
Supplement: Attachment — Submitted filename: response_to_reviewers.docx [file pgph.0004538.s005.docx]

March 2025

Marie A. Brault, PhD

Academic Editor

PLOS Global Public Health

Dear Dr. Brault,

Thank you for your review of our manuscript entitled, “*PrEP in the key population community: a qualitative study of perspectives on pre-exposure prophylaxis by gay, bisexual and other men who have sex with men and female sex workers in Kigali, Rwanda*.” We thank the reviewers for their comments, which were insightful and helpful. We have undertaken revisions in response to their suggestions and feel that these changes have substantially strengthened the manuscript. We have also addressed the editorial comments regarding style requirements, supporting information, and data availability. Below, we outline point-by-point responses to the comments by the editor and reviewers, as well as the corresponding references in the revised manuscript.

**Journal Requirements**

**1. Please include a complete copy of PLOS’ questionnaire on inclusivity in global research in your revised manuscript.**

We have included this as supplementary material.

**2. Please amend your detailed Financial Disclosure statement.**

We have amended this as requested.

**3. We have amended your Competing Interest statement to comply with journal style. We kindly ask that you double check the statement and let us know if anything is incorrect.**

We reviewed the statement and agree with the changes.

**4. Data availability**

We have clarified the possibility of publicly sharing raw data. At this time we are not able to share the full, raw data set publicly. As requested, we have replied to the editor with an email stating as much, and have amended our data availability statement as follows: Full transcripts are not publicly available as they contain sensitive information, which, though de-identified, include personal narratives that could result in identification of individuals, and because permission for public dissemination of raw data was not solicited from participants at the time of informed consent, nor from the Rwanda National Ethics Committee. De-identified data that support the findings of this study may be made available for researchers who meet the criteria for access to confidential data upon reasonable request. For data inquiries, please contact Ms. Valentine Ingabire, secretary of the Rwanda National Ethics Committee, at info@rnecrwanda.org.

**5. Please add a full list of legends for your Supporting Information files after the references list.**

We have added this at the end of the manuscript file, as requested.

**Editor**

**1. I agree with the reviewers' recommendations to ensure that person-centered language is used to refer to key populations throughout. Please also note and address the questions concerning qualitative data collection and participant recruitment, and consider whether these aspects might need to be discussed as study limitations.**

Thank you for this comment. We have updated the language and made changes, addressed questions around recruitment and data collection and updated the discussion, as detailed below.

**2. In addition to the reviewers' comments, I would encourage the authors to expand the background and introduction to provide more information on the context of key populations in Rwanda and the policy/legal environment. In the discussion, the paper references that the study city has a "relatively tolerant policy climate with respect to sexual health", but it isn't clear what that means or how that might play a role in interpreting findings, beyond being cited as a limitation. Additionally, given that the goal of qualitative studies is not generalizability, but instead an in-depth understanding of experiences and context, it isn't clear to me how this aspect would necessarily constitute a limitation.**

We appreciate these suggestions. We have expanded the introduction, moving some text from the Methods and elaborating on it to provide additional background on the policy environment with respect to LGBTI health and sex work. We agree with the editor that generalizability is not necessarily a limitation and have removed this text from the Discussion.

**3. Please include a complete copy of PLOS’ questionnaire on inclusivity in global research in your revised manuscript.**

We have included this questionnaire as an attachment / supplementary material.

**Reviewer 1**

**1. This well-executed and written study describes the qualitative barriers and facilitators of PrEP use among men who have sex with men and female sex workers in Kigali. It provides useful comparisons of different motivators and barriers, and includes an interesting discussion of social cohesion and importance of collective benefits of PrEP in this setting. It is a valuable addition to the literature and only minor edits are suggested.**

We thank the reviewer for their enthusiasm and positive feedback.

**2. Pg. 3, line 87: instead of “high-risk groups”, prioritise using people-centred language that doesn’t define people solely by their risk. E.g. “groups with higher risk of acquiring HIV”.**

Thank you for this comment. We have made the suggested change (Page 3, paragraph 5).

**3. Pg. 3, line 88: Is the number receiving PrEP medication based on having received it at least once? Are there any data on PrEP continuation that could also be cited here? Not knowing the population size estimates of MSM and FSW in Rwanda/Kigali, is there any estimate of the proportion of these groups who are on or who have started PrEP?**

We appreciate these inquiries. Although data on PrEP initiation and retention among key populations in Rwanda are fairly limited, we have added relevant text. We now provide population size estimates for FSW and GBMSM (Page 3, Paragraph 3), and further clarify that we refer to persons initiating PrEP, i.e., who received at least one prescription (Page 3, paragraph 5).

**4. Pg. 8, line 252: Is colleague the correct translation here, referring to coworker?**

Thank you. We have updated the translation to “peer,” which we believe more clearly expresses the intent of the participant (Page 8, paragraph 7).

**5. Pg. 9, line 291: Based on some of the included quotes, also implies stigma experienced around gender identity and expression.**

We agree with the reviewer, and have added text about gender identity stigma (Page 9, paragraph 6).

**Reviewer 2**

**1. This well-written manuscript presents a qualitative analysis of cisgender female sex workers and gay, bisexual, and other men who have sex with men in Kigali, Rwanda. While several findings have been previously published, and it would have been preferable to avoid the comingling of qualitative data from these two distinct populations—given that existing research highlights differences in their health experiences—the paper is noteworthy for exploring “collective antiretroviral protection” as a sexual health strategy. A strength of the study is that participants were recruited from facility and community settings and included current, former, and never PrEP users. However, the PrEP perspectives of never-users are well described in the literature; thus, including PrEP re-initiators would have been more interesting, given how users cycle on and off PrEP. Given that intersecting stigmas are recognized as a critical and complex barrier to the uptake of evidence-based HIV prevention interventions, it would have been beneficial to organize the Results (third theme) and Discussion in line with the Socio-Ecological Model, which the authors indicate they referenced in designing the interview guide. I have a few comments which the authors might find helpful.**

We appreciate this feedback from the reviewer. We agree with the reviewer that GBMSM and FSW are distinct populations with different characteristics; nonetheless, both groups face common barriers to accessing PrEP, which health systems must respond to in order to optimize care. We thus believe that presenting similarities (particularly those related to the role of community) in addition to differences between GBMSM and FSW in Rwanda is appropriate. We have also better emphasized the socio-ecological model in the Results and Discussion. We have made suggested changes as noted below in response to critiques 9 and 10.

**2. The authors state that “limited data exist describing experiences and preferences of GBMSM and FSW with respect to accessing and using PrEP” as a rationale for their study. However, a PubMed search (MSM AND PrEP AND Qualitative AND Africa) revealed 23 studies, suggesting their PrEP experiences have been amply described in the literature.**

Thank you for this comment. We clarify that the quoted text referred to GBMSM and FSW in Rwanda (Page 3, paragraph 4).

**3. Please clarify that this is a study of oral PrEP, given the availability of PrEP rings and injections as alternative formulations.**

We appreciate this comment, we have clarified this in the Introduction (Page 3, paragraph 4).

**4. Participants were recruited from June-July 2022 but interviewed from November-December 2022. What accounts for this five-month gap? Did all who indicated interest in the study eventually participate in interviews, or did some drop off or could not be reached five months later? Were all participants who were approached interviewed, or did some decline? Reasons for refusal should be described in the Results.**

We appreciate the reviewer pointing out this discrepancy. The text describing interviews in November and December 2022 was erroneous; interviews were conducted in the immediate aftermath of recruitment, also during June and July 2022. We have corrected this error in the manuscript (Page 5, paragraph 4).

**5. Methods: Line 111 refers to an interview guide. Were separate interview guides used for FSW and GBMSM? If not, this should be acknowledged as a limitation as these are distinct sub-populations.**

We appreciate this suggestion, and have noted in the Limitations that a single interview guide was used for both FSW and GBMSM (Page 12, paragraph 4).

**6. Six GBMSM reported sex with both women and men, but marital status was not reported. Were they married to women to fulfill cultural and religious norms to have children and to avoid discrimination, as often happens in this setting?**

Thank you. Unfortunately, we did not collect data from participants on marital status, and are therefore unable to discern drivers of sexual practices with respect to men and women.

**7. Results, lines 163-170: This text is unrelated to the social networks theme and can be moved to after the sentence ending on line 159.**

We agree with the reviewer, and have in fact removed most of this text (Page 6, paragraph 1).

**8. Results–Quantification of qualitative research. It is tempting to describe qualitative data using quantitative terms, e.g., “a high level of knowledge about PrEP” (how was this measured?), “a substantial number of participants,” or “tremendous amount of anticipated and enacted stigma” (how was this measured?) but this is best avoided.**

Thank you for this comment. We have removed these terms throughout the manuscript.

**9. Discussion: The authors state in the Methods that the socioecological model was used to explore individual and structural PrEP barriers and facilitators. However, this is not evident in the Results (lines 352-367), which do not appear to discuss the findings through the lens of individual, interpersonal, intersectional, or structural stigma.**

Thank you for this comment. As the reviewer notes we utilized the socioecological model (SEM) to structure data collection. We feel that the results do describe barriers to, facilitators of and experiences of PrEP engagement at multiple domains of the SEM, namely individual, interpersonal, community, structural and societal levels. We have made this more explicit in the Results and Discussion (Page 7, paragraphs 1 and 6; Page 8, paragraph 3 6-9; Page 9, sub-section title [“Multi-level stigmas…] and paragraph 2 and 6; Page 10, paragraph 1; Page 11, paragraphs 4 and 5; Page 12, paragraph 2).

**10. Discussion: Whereas stigma and discrimination in healthcare settings were described in the Results, there is no mention of gender sensitivity training for providers as a possible solution. Additionally, the paper does not mention sex work stigma (sex work is criminalized in Rwanda), particularly for GBMSM who sold sex, as per Table 1.**

We appreciate the reviewer’s comment. Participants in this highlighted the lack of training of health providers with respect to LGBTI issues; some participants advocated for additional provider training although accessing PrEP in less stigmatizing spaces was more frequently proposed as a solution. We have added text in the Results (Page 11, paragraph 1) as well as the Discussion to note provider training as another potential approach (Page 11, paragraph 5).

**11. Discussion: Generalizability – avoid the temptation to generalize findings from FSW and GBMSM in one urban setting by saying your “data suggest that offering options for PrEP care would be well received by key populations in SSA.”**

We appreciate this comment, and have modified the text to note that offering options for PrEP care “may be a promising strategy.” (Page 12, paragraph 1).

**12. The NIAID HIV Language Guide recommends not using the term MSM because it is stigmatizing. Gay, bisexual, and other men who have sex with men (GBMSM) is preferred. Moreover, Table 1 indicates that some of these men were bisexual, which speaks to the limitations of describing this population as MSM.**

Thank you for this comment. We agree with the reviewer and have changed the terminology throughout the manuscript – including the title – to GBMSM.

**13. Line 59: Clarify that the population under study is cisgender FSW and GBMSM. Currently, the reader only finds this out after seeing sex at birth data in Table 1.**

We appreciate this point, and have added clarifying text in the Abstract (Page 2) and Introduction (Page 3, paragraph 1).

**14. Lines 87-88: FSW and MSM were defined in the Introduction and do not need to be written in full in the Methods.**

We have removed the full text as suggested (Page 3, paragraph 2).

**15. Lines 148-149: Please provide a citation for the COREQ guidelines.**

Thank you for this comment, we have provided a citation as suggested (Page 5, paragraph 3)

**16. Line 382: The preferred term is “serodifferent” not serodiscordant.**

We have made the suggested change (Page 3, paragraph 4 and Page 12, paragraph 2).

**Reviewer 3**

**1. In qualitative research, the interviewer is the instrument. You have mentioned that three researchers were trained (?) on qualitiative research. Those interviewers need to be well versed with exploration and probing of issues and potential themes.**

Thank you for this comment. We have elaborated on the interviewers’ backgrounds in this area, noting that they all have both clinical and research experience working with the study population and received specific training on use of the interview guide for this study (Page 4, paragraph 4).

**2. I have concerns about the emerging themes. Who decided to stop at this number of participants? Generally, we are looking for saturation in emerged themes before we decide to stop further recruitment. I cannot see any discussion about this.**

We appreciate the reviewer’s question. Based on study aims and available resources, we initially planned for a sample of approximately 30 participants, considered a reasonable estimate for achieving saturation in a sample (Baker, et al, National Centre for Research Methods Review Paper, 2012). While the logistics of conducting the study (i.e. need to conduct the analysis in English, duration of time needed for interview translation, and the geographic separation of the study team) limited our capacity to assess data saturation during the data collection process, regular discussions during the coding and analysis established that data saturation had been reached, as we now note in the text (Page 5, Paragraph 2).

**3. Would be good if you present information about recruitment- i.e. how many approached and how many refused to participate. This will give an additional idea about enrolled individuals- as you highlighted mught be very motivated. You also need to present information about recruitment of individuals from each recruitment centre, the three medical centre and 7 community centres.**

Thank you for this comment. Unfortunately, we do not have access to this information. We worked with staff at health centers and associations to identify potential participants. The research team only received contact information for people who were deemed eligible by health centers/association staff and interested. During the screening process, all 32 persons were found to be eligible for the study, and all agreed to participate.

**Reviewer 4**

**1. Overall, the paper is clear and well-written and was enjoyable to read.**

We thank the reviewer for this positive feedback.

**2. Section that states that current care models are not optimized for KPs can be expanded to be more descriptive of what current care models there are and why they are not working well, or describe that they are tailored towards other risk groups. The evidence described on factors does not explicitly speak to this. But this implies that PrEP services are not differentiated to the population, for example primarily being clinic/health facility based. But some description of this and references would be helpful.**

Thank you for this suggestion. We have added text in the Introduction (Page 3, paragraph 2) and Methods (Page 4, paragraph 1) highlighting why the current model of care is suboptimal for KPs and noting that care is not differentiated.

**3. Suggest including a citation for the government of Rwanda initiation of PrEP for KPs (first line of 3rd paragraph)**

We have added a citation as suggested (Page 3, paragraph 4, Ref 32)

**4. For context for the reader, in the background or methods, could indicate if same-sex sexual activity is considered legal in Rwanda, as well as if sex work is legal or criminalized in the country. This may be influential in terms of how individuals access care/stigma felt, etc. and this information would be helpful for the reader.**

We appreciate this suggestion. We have added text in the Introduction describing the current policy/legal climate regarding same-sex sexual activity and sex work, noting that these are decriminalized but not broadly accepted (Page 3, paragraph 3).

**5. Is there any population estimate for FSW and MSM – in Rwanda overall, or in Kigali? This will be helpful, as list the number of individuals on PrEP as of June 2023.**

Thank you for this comment. We have added population estimates for GBMSM and FSW (Page 3, paragraph 3).

**6. FSW and MSM are spelled out in paragraph 2 of the Methods – as acronym has already been used, can use consistently throughout paper**

We have removed the indicated text (Page 3, paragraph 5).

**7. In the methods, it is indicated that individuals were purposefully recruited to include those that were using PrEP, had previously used PrEP, and those that had never used PrEP. Is there a eligibility screening tool that was used that can be provided as part of the supplementary materials? The in-depth interview guide provided asks questions including ‘Have you ever heard of PrEP?’, but to be recruited, it seems like this question would already have been asked/known? Same with have you ever used PrEP question.**

Thank you for this comment. We have now provided additional detail about recruitment in the Methods (Page 4, paragraph 3). Research staff met with providers at health centers and associations to present information about the study, after which clinical/association staff were asked to identify and refer potential participants on PrEP, who had stopped PrEP, and who had been evaluated for but never initiated PrEP. Individuals were then screened by research staff based on inclusion criteria: 1) HIV-negative by self-report; 2) self-identify as GBMSM or FSW; 3) at least 18 years of age. As we purposefully recruited individuals who had never used PrEP, we did not make awareness of PrEP or prior use of PrEP part of the eligibility criteria.

**8. Please review the interview guide provided. In one place it indicates in Section II, Question 2, have you ever used PrEP, if no, ask 2a, and skip to Section II. I think this means skip to Section III. Please review and revise as needed.**

The reviewer is correct. We have changed this from II to III (Interview guide).

**9. The time between recruitment and interviews was long (several months). Were any individuals recruited lost to follow-up because of this delay between recruitment and interview?**

Thank you for this comment. As noted above (Reviewer 2, critique 4) we have corrected the text to reflect that recruitment and data collection occurred in proximity to each other (Page 5, paragraph 4).

**10. Spell out LGBTI at first use**

We have made this change (Page 3, paragraph 3).

**11. There are a number of yes/no questions in the interview guide – have you considered adding these to the results?**

Thank you for this comment. We have the most relevant of these data in Table 1.

**12. Additional quotations from participants in the stigma section of the results could be beneficial. In particular, in this section there is not a quote from a FSW.**

We appreciate the reviewer’s suggestion and have added a quote from a female sex worker in the stigma sub-section of the results (Page 9, paragraph 3).

**13. Additional quotes in the community access results section would be beneficial to the paper.**

Thank you for this suggestion. We have added a quote in this subsection from a participant advocating for additional LGBTI sensitivity training for health center staff (Page 11, paragraph 2).

**14. Overall, the FSW point of view in the results seems limited. Were there any differences by KP group in terms of how delivery of PrEP is preferred (i.e., in the community or at a health center, delivery at home by a community health care workers…?). I see now that this is mentioned in a paragraph in the discussion – emphasizing the importance of differentiated care, however, it would be beneficial to include these findings in the results, as that is not clear currently.**

We appreciate this suggestion. We have added text in the Results (Page 10, paragraph 5 and Page 11, paragraph 1) to emphasize observed differences.

**15. Some of the probes used in the interview guide seem leading – i.e., biggest challenges accessing PrEP. Did the probes influence the participants’ responses in any way? Is this a potential limitation to add?**

Thank you for this comment. We do not believe that the interview guide overly influenced participant responses. Questions were asked open-endedly to allow participants to highlight personal factors impacting PrEP delivery. Probes then focused on specific factors highlighted in the PrEP literature, to allow participants an opportunity to provide their perspective on these particular areas. We have added a sentence in the Methods (Page 4, paragraph 4) noting this.

**16. As the study made sure to include individuals who were currently on PrEP, had stopped PrEP, had never started PrEP, were the data analyzed by these disaggregations in any way?**

We appreciate this comment. We did examine differences by those who continued, stopped or never started PrEP. While there were some differences between these groups with respect to knowledge about PrEP or motivation to use it, we did not observe major differences with respect to the larger themes that ultimately emerged from the analysis.

Thank you for your continued interest and for considering this manuscript for publication in *PLOS ONE.* We very much appreciate the reviewers’ thoughtful critiques and feel that their comments have significantly improved this manuscript. We look forward to hearing back from you.

Sincerely,

Jonathan Ross MD, MS
